# Salivary and serum asprosin hormone levels in the 2018 EFP/AAP classification of periodontitis stages and body mass index status: a case-control study

**DOI:** 10.1007/s00784-024-05494-9

**Published:** 2024-01-13

**Authors:** Sema Nur Sevinç Gül, Didem Özkal Eminoğlu, Esra Laloğlu, Tuğba Aydın, Alparslan Dilsiz

**Affiliations:** 1 Department of Periodontology, Faculty of Dentistry, Atatürk University, Erzurum, Turkey; 2 Department of Medical Biochemistry, Faculty of Medicine, Atatürk University, Erzurum, Turkey

**Keywords:** Adipokines, Body Mass Index, Oral medicine, Periodontitis, Periodontal diseases

## Abstract

**Objectives:**

A newly discovered adipokine known asprosin in serum and saliva in patients with periodontitis has not been explored. The aim of this study was to determine the relationship between serum and saliva asprosin levels and periodontitis by grouping it according to body mass index (BMI).

**Materials and methods:**

The study was conducted on 65 systemically healthy patients (35 patients with periodontitis (periodontitis group), 30 periodontally healthy patients (control group)). In each patient, age, BMI, and clinical periodontal parameters (plaque index (PI), gingival index (GI), probing depth (PD), and clinical attachment level (CAL)) were evaluated. Statistical analyses were conducted utilizing the Student *t*-test, ANOVA, and Pearson correlation analysis. For the significance level of the tests, *p*<0.05 were accepted.

**Results:**

The serum and saliva were collected to assess asprosin levels. Both the serum and saliva asprosin levels were statistically significantly higher in the periodontitis group than in the control group (*p*<0.001). Saliva and serum asprosin levels were directly proportional to the severity of the periodontal disease (*p<0.05*). Asprosin levels were higher in patients with a higher BMI (*p<0.05*).

**Conclusion:**

Asprosin levels were increased in periodontitis, and even a high BMI status apparently affected the levels of this hormone. It is thought that asprosin may be a useful biomarker in evaluating the relationship between periodontal status and BMI.

**Clinical relevance:**

Asprosin may be a useful parameter as a biomarker of periodontal disease progression. However, BMI status should be considered when evaluating asprosin levels in patients with periodontitis.

## Introduction

Periodontal disease is a chronic, multifactorial, and infectious disease caused by bacteria. It is characterized by the formation of an inflammatory response in the supporting bone and connective tissue against microbial dental plaque, and the nature of the resulting inflammatory response determines the course of periodontal disease [1, 2]. Although periodontitis is a disease that develops in response to bacteria and their products, the course of the disease is regulated by the host tissue response. Periodontal tissue may act as a source of endocrine-like inflammatory mediators (such as IL-1β, TNF-α, and IL-6) that are important for treating periodontal inflammation and can affect glucose and lipid metabolism [3]. According to current study, Body Mass Index (BMI) was correlated with clinical attachment loss (CAL), pocket depth (PD), plaque index (PI), stage and grade of periodontitis, and number of remaining teeth [4].

Adipose tissue, particularly white adipose tissue, is an endocrine organ in which a number of pro- or anti-inflammatory cytokines known as adipokines are produced, including adiponectin, visfatin, leptin, and resistin [5, 6]. Additionally, periodontal diseases may have an effect on adipokines or on the periodontal response [7]. Asprosin, a recently discovered glucogenic adipokine, is produced by the C-terminal cleavage product of profibrillin coded by profibrillin gene (FBN1), is mainly synthesized by white adipose tissue and released during fasting [8]. Asprosin circulates in the blood at nanomolar levels and is taken to the liver, where it triggers the G protein-cAMP-PKA pathway, causing rapid glucose delivery into circulation [9]. Asprosin is associated with diseases such as diabetes mellitus, obesity, polycystic ovary syndrome, and cardiovascular diseases [10]. Recent research has revealed that asprosin is implicated in many cell types’ inflammatory responses. According to the TLR4-JNK pathway, asprosin causes pancreatic islet cells to become inflamed and dysfunctional [11]. Moreover, asprosin stimulates endoplasmic reticulum stress-induced skeletal muscle inflammation in a PKC-dependent way [12]. We thought that periodontitis might have an impact on the level of circulating asprosin because of its systemic effects (similarly to other chronic inflammatory diseases). As far as the study’s authors are aware, no study could be found that investigated the relationship between periodontitis and asprosin, a hormone that may be helpful in the approach to periodontology as an additional biochemical marker to monitor the periodontal status and BMI of patients.

This study aimed to (I) investigate levels of serum and saliva asprosin in patients with periodontitis (II) determine whether asprosin levels were related to clinical periodontal parameters, and (III) investigate the relationship between serum and saliva asprosin levels and periodontitis by grouping it according to BMI.

## Methods

Sixty-five systemically healthy individuals were recruited from the Clinic of Periodontology, Faculty of Dentistry, Atatürk University, between October 2021 and July 2022. The study population was designated into two groups: 1. Thirty-five patients with periodontitis (periodontitis group), 2. Thirty periodontally healthy patients (control group). This study was approved by the Institutional Internal Review and Ethics Board (AU-IIREB reference code: 511) and conducted according to the 2008 Declaration of Helsinki and its later amendments. Registration number (NCT05449821) was taken from ClinicalTrials.gov website. A signed consent form was obtained from all participants prior to the commencement of the study.

The inclusion criteria of the study were as follows: Participants were aged between 18 and 60 years old, were generally healthy, non-smoking, and none had undergone periodontal therapy and/or antibiotic therapy in the past 6 months [13]. Patients with systemic diseases, such as diabetes mellitus, rheumatoid arthritis, and cancer, pregnant or breastfeeding women were excluded [14].

### Clinical examination

Complete clinical periodontal examination was performed by a single-blinded examiner (DÖE) using Williams probes (Williams, Hu-Friedy, Chicago, IL, USA). The periodontal parameters probing depth (PD), clinical attachment level (CAL), plaque index (PI)[15], gingival index (GI) [16] and bleeding on probing (BOP) were all measured during a full-mouth clinical examination of all patients. The measurements were recorded from 6 sites (disto-buccal, mesio-buccal, mid-buccal, and disto-palatal, mesio-palatal, mid-palatal). A sample group of 10 people with periodontitis who rejected to take part in the study served as the intra-examiner calibration sample prior to the study’s start. Within 24 h, each patient’s CAL and PD were measured twice at six different sites on each tooth. After the assessments achieved the established success criteria of CAL and PD (the percentage of agreement within 1 mm between repeated measurements should be at least 90%), they were considered to be reproducible. The BMI values of the participants were used using an electronic scale (Charder MS-3400-1, Taiwan) that can give height, weight and BMI values, and the method of dividing the weight by the square of the height was used [17].

### Study groups

Using the following criteria, the cases were allocated to the study groups:

- Control group: The sites presented BOP < 10% and PD ≤ 3 mm, no sites had attachment loss. There was no evidence of alveolar bone loss on radiographs or a history of periodontitis.
- Periodontitis group: At least two non-adjacent teeth showed signs of interdental CAL or buccal or oral CAL ≥ 3 mm and PD >3 mm. In line with the periodontal parameters obtained, it was divided into stages according to the classification conducted by the 2018 EFP and AAP [17].
- Sample Collection

During the collection of the serum samples, blood samples were taken from the antecubital fossa while the patients remained in a sitting position for the purpose of standardization. Blood samples taken for biochemistry tests were kept for 20–30 min for coagulation. The tube was centrifuged at +4°C and 4000 rpm for 15 min while remaining upright [18]. The collected serum samples were aliquoted, stored until analysis day at -80° in a deep freezer.

Between the hours of 9:00 AM and 10:00 AM, whole saliva samples were taken from the patients and controls and placed into weighted 5-ml sterile polypropylene tubes for 10 min. Ambient conditions were provided for all participants to sit comfortably in a resting position. For two hours prior to collection, no oral stimulation was allowed in order to rule out the influence of mastication or eating. During this time, the sitting patients collected their saliva, gathered it at the back of the mouth, and occasionally emptied it into a collection tube. For 5 minutes, the samples were centrifuged at 10,000 rpm [19]. The final supernatant was kept at -80°C until it was time to use it.

### Biochemical analysis

As directed by the manufacturer, the serum samples were examined using a “Human Asprosin ELISA Kit.” (BT LAB, Cat. No. E4095 Hu, China). This kit has a detection range of 0.5–100 ng/mL. This test has a sensitivity of 0.23 ng/mL. The kit manufacturer specifies that the asprosin measurement’s interassay and intraassay coefficients of variation are < 10% and <8%, respectively. In a summary, each sample and standard (128, 64, 32, 16, 8, and 4 ng/mL) were introduced to the appropriate well that had already been coated with human asprosin antibodies. The antibodies that were coated on the wells bound the asprosin that was present in the sample. After that, human asprosin antibodies that had been biotinylated were added and bound to the asprosin in the sample. The biotinylated asprosin antibodies were then combined with streptavidin-HRP and bound to them. Unbound streptavidin-HRP was removed following incubation. The substrate solution was then added, and the color of the mixture changed in direct proportion to the level of human asprosin present. By adding an acidic stop solution, the process was stopped, and absorbance at 450 nm was measured. The optical density (OD) of the asprosin samples was compared to the standard curve to estimate their levels [20].

### Statistical analyses

The results were described as the mean ± standard deviation (SD). The normal distribution suitability of the parameters was determined using Kolmogorov-Smirnov tests. Since the asprosin values of the healthy and test groups were normally distributed, Student’s *t-*test was used to compare the asprosin levels of the two groups. BMI comparisons of the control and periodontitis groups were performed on an independent sample using Student’s *t*-test. The stage of the periodontitis group was analyzed statistically using One-way ANOVA. Also, analysis of covariance (ANCOVA) was used to investigate the effect of the body mass index as covariates. Pearson’s correlation test was also performed. ROC curve analysis was used to determine the discriminating power of asprosin in the diagnosis of periodontitis. The value of *p < 0.05* was statistically significant. Statistical analyses were performed by using the SPSS 20.0 statistical software program (SPSS Inc., Chicago, IL, USA).

## Results

Sixty-five patients were included in this study, and there was no statistically significant difference between the groups in terms of gender (*p*=0.97). Asprosin levels and other factors were compared between participants with periodontitis and those with control (Table 1). Both the serum and saliva asprosin levels were statistically significantly higher in the periodontitis group than in the control group (*p*<0.001). Saliva asprosin and serum asprosin levels were statistically significantly different in the periodontitis group (*p*<0.001). Saliva asprosin and serum asprosin levels were statistically significantly different in the control group (*p*=0.04).

**Table 1:** Comparison between control and periodontitis groups

	Control Group (*n*=30) Mean ± SD	Periodontitis Group (*n*=35) Mean ± SD	*p*
*Salivary Asprosin (ng/mL)*	11.59±10.03	48.18±12.30	<0.001
*Serum Asprosin(ng/mL)*	17.66±11.84	72.38±33.59	<0.001
*BMI (kg/m*^*2*^*)*	21.06±3.45	23.55±4.54	0.017
*Age (years)*	33.63±11.38	38.23±6.95	0.05
*PI*	0.27±0.45	1.86±0.65	<0.001
*GI*	0±0	1.71±0.62	<0.001
*BOP (%)*	0±0	76.34±12.69	<0.001
*CAL (mm)*	0±0	5.29±2.92	<0.001
*PD (mm)*	2.63±0.49	5.83±2.22	<0.001

Abbreviations: *BMI* body mass index, *PI* plaque index, *GI* gingival index, *BOP* bleeding on probing, *CAL* clinical attachment level, *PD* pocket depth, *SD* standart deviation

In the periodontitis group, when classified as stage I, II, III and IV according to the 2018 EFP/ AAP classification, serum asprosin levels are respectively; 47.48 ± 2.19, 57.27 ± 8.21, 82.30 ± 24.67, 124.18 ± 38.43 and saliva asprosin levels are respectively; 34.91±5.23, 46.01±3.23, 54.50±2.07, 64.41±14.27. A statistically significant difference was found between both serum and saliva asprosin levels in the Stage IV periodontitis group compared to the Stage I and II periodontitis groups (*p*<0.001) (Table 2).

**Table 2:** The distribution of asprosin levels according to the stage of periodontitis group

	Salivary Asprosin Levels	Serum Asprosin Levels
Stage I (n:10)	34.91±5.23	47.48 ± 2.19
Stage II (n:10)	46.01±3.23	57.27 ± 8.21
Stage III (n:9)	54.50±2.07	82.30 ± 24.67
Stage IV (n:6)	64.41±14.27	124.18 ± 38.43
I vs II	*p*<0.01	*p*<0.01
I vs III	*p*<0.001	*p*<0.01
I vs IV	*p*<0.001	*p*<0.001
II vs III	*p*<0.01	*p*<0.01
II vs IV	*p*<0.001	*p*<0.001
III vs IV	*p*<0.01	*p*<0.01

The distribution of asprosin levels according to BMI in the periodontitis group are shown in Table 3. BMI was significantly higher in the periodontitis group than in the control group (*p*=0.017). A strong positive and significant correlation was found between the asprosin levels and BMI index (*r*=0.77, *p*<0.001). Also, in an ANCOVA taking BMI as covariance the difference between periodontitis and control group level remained significant for saliva and serum asprosin level, (F: 161;61, respectively, *p*<0.001).

**Table 3:** The distribution of asprosin levels according to BMI in the periodontitis group

	Salivary Asprosin Levels	Serum Asprosin Levels
Normal (Group 1, n:15)	37.75±6.05	49.31±3.45
Overweight (Group 2, n:12)	51.92±3.38	65.74±13.28
Obese (Group 3, n: 8)	62.13±12.80	125.59±27.01
Statics	*p*	*p*
1 vs 2	*p*<0.001	*p*<0.05
1 vs 3	*p*<0.001	*p*<0.001
2 vs 3	*p*<0.05	*p*<0.001

The correlation of saliva and serum asprosin levels with other variables are shown in Table 4. There was a significant and positive correlation between the serum and saliva asprosin levels and PI, GI, BOP, CAL, and PD (*p*<0.001). The correlation between the asprosin levels with CAL and PD was the strongest. There was a significant relationship between the serum and saliva asprosin levels *r: 0.663*, *p*<0.001.

**Table 4:** Pearson correlation of serum and saliva asprosin levels with other variables

		Serum Aspirosin	Saliva Asprosin
PI	***r***	0.676^**^	0.661^**^
	***p***	<0.001	<0.001
GI	***r***	0.726^**^	0.760^**^
	***p***	<0.001	<0.001
BOP	***r***	0.715^**^	0.804^**^
	***p***	<0.001	<0.001
CAL	***r***	0.885^**^	0.793^**^
	***p***	<0.001	<0.001
PD	***r***	0.882^**^	0.760^**^
	***p***	<0.001	<0.001
Serum Asprosin	***r***		0.663^**^
	***p***		<0.001

Abbreviations: *PI* plaque index, *GI* gingival index, *BOP* bleeding on probing, *CAL* clinical attachment level, *PD* pocket depth

**Fig. 1:**
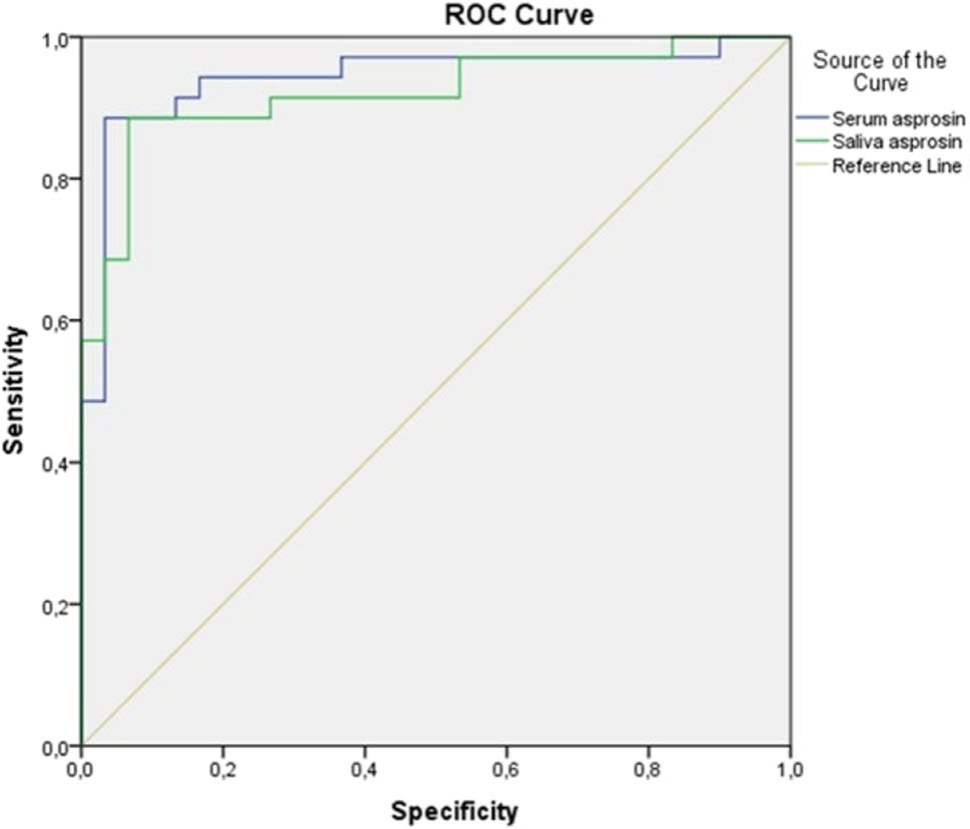
Determination of the diagnostic sensitivity and specificity of serum and saliva asprosin levels in periodontitis and control groups by ROC curve analysis. ROC: receiver-operating characteristic curve

When the serum cut-off value was 25.36, the sensitivity was 94% and the specificity was 83% (AUC = 0.94, *p* < 0.001) (95% confidence interval, 0.88–1.0). When the saliva cut-off value was 19.81, the sensitivity was 89% and the specificity was 80% (AUC = 0.92, *p* < 0.001) (95% confidence interval, 0.85–0.99). The ROC curve is shown in Fig. 1.

## Discussion

Asprosin was first identified by Romere et al., who also verified that patients with neonatal progeroid syndrome have mutations at the 3' terminus of the fibrillin-1 (FBN-1) gene that cause asprosin to be absent near the C-terminal cleavage site [8]. As a result of this study, it was found that both serum and saliva asprosin levels were higher in the periodontitis group compared to the control group. Based on the results of a literature search, it was concluded that this study is one of the first of its kind to investigate the serum and saliva asprosin levels of periodontitis patients.

Asprosin has been demonstrated to bind to hepatocyte membranes and stimulate glucose synthesis through the cyclic AMP (cAMP)-protein kinase A (PKA) pathway [8]. According to a different study, asprosin affects mice through the olfactory receptor OLFR734 that is a G-protein coupled receptor [21]. Li et al. demonstrated that asprosin therapy significantly decreased circulating glucose levels in OLFR734-knockdown mice compared to their wild-type counterparts. Additionally, asprosin enhanced circulation in response to fasting raised hepatic cAMP levels and glucose synthesis, however these effects were reportedly less pronounced in the OLRF734-knockdown mice [22]. The role of asprosin in the inflammatory response in different cell types has been detailed in a number of recent research. According to research, asprosin activates the TLR4-JNK pathway, which results in inflammation and malfunction of pancreatic islet cells [11]. Additionally, in vivo studies showed that asprosin increased endoplasmic reticulum stress, glucose intolerance, insulin resistance, and the flow of pro-inflammatory cytokines (monocyte chemoattractant protein-1, IL-6, and TNF) [23]. Huang et al. reported that asprosin has a proinflammatory effect on the vascular endothelium, and also alleviates vascular endothelial inflammation caused by high-fat diet by neutralizing it with asprosin antibody. Asprosin has been reported to induce and exacerbate vascular endothelial inflammation through the IKKβ-NF-κBp65 pathway [24].Asprosin induced a pro-inflammatory response in THP-1 macrophages, as shown by Shabir et al, by greatly increasing the production and secretion of important pro-inflammatory mediators such TNF, IL-1, IL-8, and IL-12 [12].

Periodontitis is a chronic inflammatory condition that produces an inflammatory and immune response through the interaction of bacterial products and multiple cell populations [25]. Since periodontitis is a chronic inflammatory disease, asprosin levels in both serum and saliva were higher in the periodontitis group compared to the control group. We considered that periodontitis might have an impact on the level of circulating asprosin because of its systemic effects (similarly to other chronic inflammatory diseases). Accordingly, asprosin may be a biomarker in detecting periodontitis and linking periodontitis with BMI status. In this study, periodontitis was staged, and it was investigated whether asprosin levels were affected according to the severity of the disease. When the serum asprosin levels were analyzed, a statistically significant difference was found between stages II and IV and between stages I and IV (*p*<0.001). Accordingly, as the severity of periodontitis increased, the serum asprosin level increased. The increase in serum asprosin level according to the severity of periodontitis revealed the relationship between the severity of inflammation and asprosin levels. Additionally, asprosin levels had a significant and positive correlation with PI, GI, BOP, CAL, and PD. The correlation between asprosin levels with CAL and PD was the strongest. Periodontal disease severity and degree of damage are related to CAL and PD. The positive correlation of asprosin with clinical indicators suggests that this marker can be used to determine the severity of periodontal disease.

Asprosin and BMI index showed a substantial positive and significant association in this investigation. Uğur and Aydın reported that asprosin levels in serum and saliva increased with BMI, which is consistent with our study [26]. However, in addition to this study, it was determined by the help of ANCOVA analysis that there was no confounding factor of BMI with high asprosin levels in the periodontitis group in our study and that this disease was a result of periodontitis. Additionally, a number of clinical trials revealed that people who were overweight or obese had significantly higher serum asprosin levels [27–29]. Circulating asprosin was found to cross the blood-brain barrier and directly activate orexigenic AgRP+ neurons through a cAMP-dependent pathway in a study. It was also reported to trigger appetite and boost body weight by inhibiting anorexigenic proopiomelanocortin (POMC)-positive neurons in a downstream manner in a GABA-dependent manner [9]. In humans, a genetic deficiency in asprosin causes NPS, which is characterized by anorexia and extreme weakness [8]. However, there is conflicting information on the levels of circulating asprosin in obese kids. Children with obesity had considerably lower serum levels of asprosin than children of normal weight, according to studies by Long et al. and Corica et al. [30, 31]. Additionally, Silistre and Hatipoglu discovered no gender-related changes in the asprosin levels in the blood of children with obesity compared to the normal weight group [32].

Many studies have reported that obesity and overweight are associated with the risk of periodontitis [33, 34]. The reason for this relationship may be the involvement of proinflammatory cytokines secreted from adipose tissue in the formation of excessive inflammatory response in periodontitis [35]. It has been shown that the relationship between obesity and periodontitis is bidirectional, and the presence of inflammation in periodontal tissues may be a predisposing factor for obesity [36]. As with obesity, adipokines and systemic low-grade inflammation may explain another mechanism by which periodontitis is associated with obesity. It has been suggested that obesity may contribute to periodontitis through altered adipokine levels [37]. Since there is no study of asprosin, a recently discovered adipokine, with periodontitis, there is no study in which we can compare the relationship of serum and saliva with BMI in individuals with periodontitis.

One of the limitations of this study was that the gender and age factors were not limited. The levels of adipokine hormones may differ between different genders and age groups. Age and gender standardized-controlled trials need to obtain precise data. Another limitation of the study was examining the serum and saliva asprosin levels using a limited sample size. For the objective of evaluating activity, it may be helpful to compare the levels of asprosin in the serum and saliva with those in GCF and gingival tissue (as well as research at how periodontal treatment affects asprosin levels) in patients with various types and degrees of periodontitis.

## Conclusion

As a result of our study, asprosin may be a useful parameter as a biomarker in the course of periodontal diseases. However; BMI status should be considered when evaluating asprosin levels in patients with periodontitis. It is necessary to conduct molecular research to determine the function of asprosin in the inflammatory pathway in light of the pathophysiology of periodontal tissues.

## References

[1] Loesche WJ, Grossman NS (2001). Periodontal disease as a specific, albeit chronic, infection: diagnosis and treatment. Clin Microbiol Rev.

[2] Cobb CM, Williams KB, Gerkovitch MM (2009). Is the prevalence of periodontitis in the USA in decline?. Periodontol 2000.

[3] Grossi SG, Genco RJ (1998). Periodontal Disease and Diabetes Mellitus: A Two-Way Relationship. Ann Periodontol.

[4] Çetin MB, Sezgin Y, Önder C, Bakirarar B (2022). The relationship between body mass index and stage/grade of periodontitis: a retrospective study. Clin. Oral Investig.

[5] Dilsiz A, Kiliç N, Aydin T, Nesibe Ates F, Zihni M, Bulut C (2010). Leptin levels in gingival crevicular fluid during orthodontic tooth movement. Angle Orthod.

[6] Zhu J (2017). Association of circulating leptin and adiponectin with periodontitis: A systematic review and meta-analysis. BMC Oral Health.

[7] Sun WL, Chen LL, Zhang SZ, Wu YM, Ren YZ, Qin GM (2011). Inflammatory cytokines, adiponectin, insulin resistance and metabolic control after periodontal intervention in patients with type 2 diabetes and chronic periodontitis. Intern Med.

[8] Romere C (2016). Asprosin, a Fasting-Induced Glucogenic Protein Hormone. Cell.

[9] Duerrschmid C (2017). Asprosin is a centrally acting orexigenic hormone. Nat Med.

[10] Yuan M, Li W, Zhu Y, Yu B, Wu J (2020). Asprosin: A Novel Player in Metabolic Diseases. Front. Endocrinol. (Lausanne).

[11] Lee T, Yun S, Jeong JH, Jung TW (2019). Asprosin impairs insulin secretion in response to glucose and viability through TLR4/JNK-mediated inflammation. Mol. Cell Endocrinol.

[12] Shabir K (2023). Asprosin Exerts Pro-Inflammatory Effects in THP-1 Macrophages Mediated via the Toll-like Receptor 4 (TLR4) Pathway. Int J Mol Sci.

[13] Isler SC (2021). Evaluation of adipokines and inflammatory mediator expression levels in patients with periodontitis and peri-implantitis: a cross-sectional study. Clin Oral Investig.

[14] Borilova Linhartova P (2019). Adipokine gene variability and plasma levels in patients with chronic periodontitis -a case-control study. Braz Oral Res.

[15] Silness J, Löe H (1964). Periodontal disease in pregnancy. II. Correlation between oral hygiene and periodontal condtion. Acta Odontol Scand.

[16] Löe H, Silness J (1963). Periodontal disease in pregnancy. I. Prevalence and severity. Acta Odontol Scand.

[17] Tonetti MS, Greenwell H, Kornman KS (2018). Staging and grading of periodontitis: Framework and proposal of a new classification and case definition. J Periodontol.

[18] Cantay H, Binnetoglu K, Gul HF, Bingol SA (2022). Investigation of serum and adipose tissue levels of asprosin in patients with severe obesity undergoing sleeve gastrectomy. Obesity.

[19] Özcan E, Saygun NI, Serdar MA, Kurt N (2015). Evaluation of the salivary levels of visfatin, chemerin, and progranulin in periodontal inflammation. Clin Oral Investig.

[20] Laboratory BT (2021). Human Asprosin ELISA kit.

[21] Li E (2019). OLFR734 Mediates Glucose Metabolism as a Receptor of Asprosin. Cell Metab..

[22] Li E (2019). OLFR734 Mediates Glucose Metabolism as a Receptor of Asprosin. Cell Metab.

[23] Jung TW (2019). Asprosin attenuates insulin signaling pathway through PKCδ-activated ER stress and inflammation in skeletal muscle. J Cell Physiol.

[24] Huang Q et al (2022) Asprosin Exacerbates Endothelium Inflammation Induced by Hyperlipidemia Through Activating IKKβ-NF-κBp65 Pathway. Inflammation:1–16. 10.1007/S10753-022-01761-7/FIGURES/8 10.1007/s10753-022-01761-7 36401667

[25] Yucel-Lindberg T, Båge T (2013) Inflammatory mediators in the pathogenesis of periodontitis. Expert Rev Mol Med 15. 10.1017/ERM.2013.8 10.1017/erm.2013.8 23915822

[26] Ugur K, Aydin S (2019) Saliva and blood asprosin hormone concentration associated with obesity. Int J Endocrinol 2019. 10.1155/2019/2521096 10.1155/2019/2521096 PMC6458951 31049060

[27] Ju X et al (2014) IL-6 regulates extracellular matrix remodeling associated with aortic dilation in a fibrillin-1 hypomorphic mgR/mgR mouse model of severe Marfan syndrome. J. Am. Heart Assoc. 3(1). 10.1161/JAHA.113.000476 10.1161/JAHA.113.000476 PMC3959679 24449804

[28] Wang CY (2018). Serum asprosin levels and bariatric surgery outcomes in obese adults. Int. J. Obes. 2018 435.

[29] Ceylan Hİ, Saygın Ö, Özel Türkcü Ü (2020) Assessment of acute aerobic exercise in the morning versus evening on asprosin, spexin, lipocalin-2, and insulin level in overweight/obese versus normal weight adult men. 37(8):1252–1268. 10.1080/07420528.2020.1792482 10.1080/07420528.2020.1792482 32741294

[30] Long W (2019). Decreased Circulating Levels of Asprosin in Obese Children. Horm Res Paediatr.

[31] Corica D (2021). Asprosin serum levels and glucose homeostasis in children with obesity. Cytokine.

[32] Sünnetçi Silistre E, Hatipoğl HU (2020). Increased serum circulating asprosin levels in children with obesity. Pediatr Int.

[33] Haffajee AD, Socransky SS (2009). Relation of body mass index, periodontitis and Tannerella forsythia. J Clin Periodontol.

[34] Suvan JE (2015). Association between overweight/obesity and increased risk of periodontitis. J Clin Periodontol.

[35] Ritchie CS (2007). Obesity and periodontal disease. Periodontol. 2000.

[36] Boesing F, Patiñeo JSR, Da Silva VRG, Moreira EAM (2009). The interface between obesity and periodontitis with emphasis on oxidative stress and inflammatory response. Obes Rev.

[37] Zhang L et al (2014) Adiponectin ameliorates experimental periodontitis in diet-induced obesity mice. PLoS One 9(5). 10.1371/JOURNAL.PONE.0097824 10.1371/journal.pone.0097824 PMC4023953 24836538

